# Supplementary figures and images for: Proteome-wide analysis of CD8+ T cell responses to EBV reveals differences between primary and persistent infection
Source: PLoS Pathog. 2018 Sep 24;14(9):e1007110. doi: 10.1371/journal.ppat.1007110 (PMC6171963; doi:10.1371/journal.ppat.1007110)

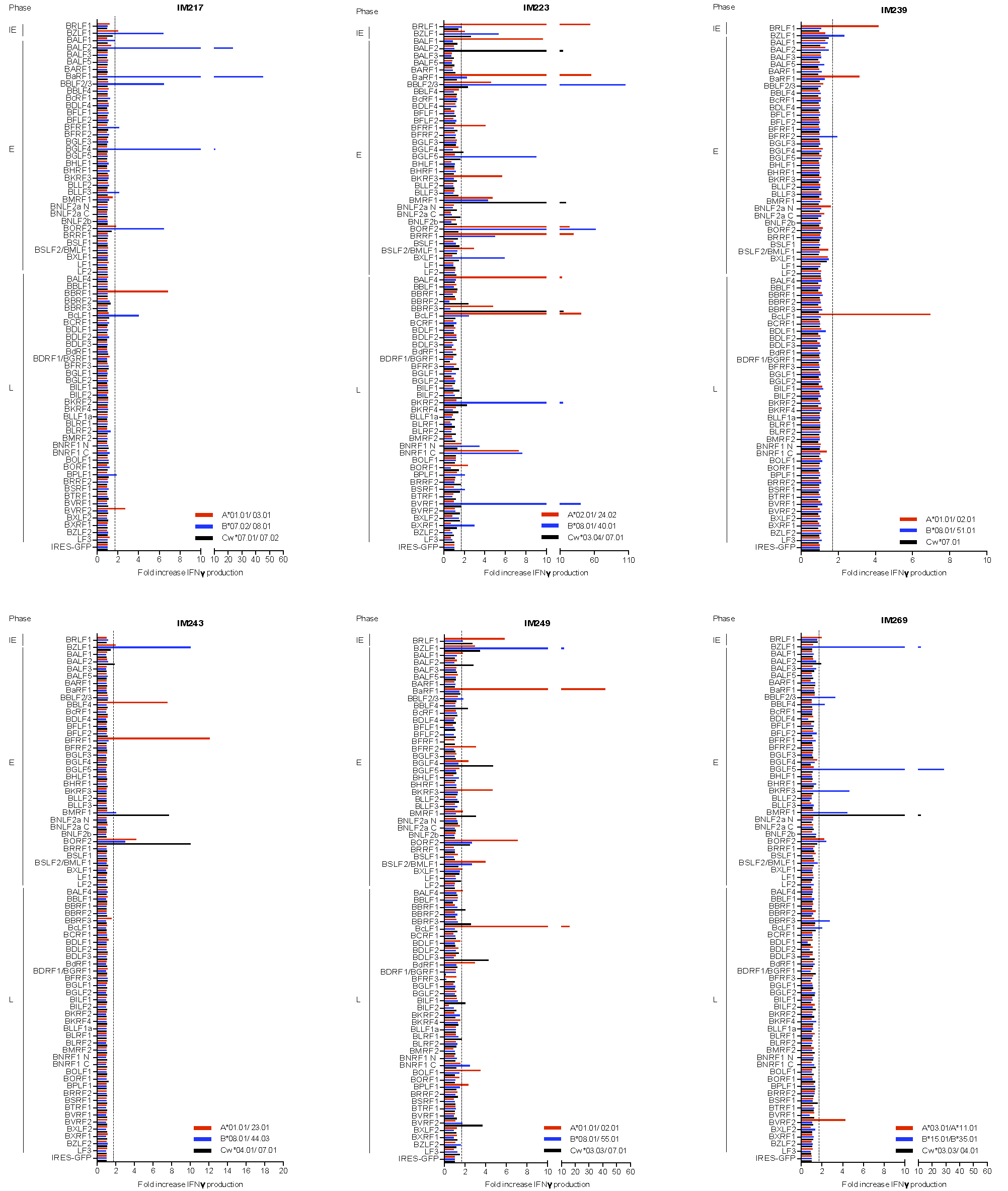

Supplement: S1 Fig — Data are presented as in Fig 2. (TIF) [file ppat.1007110.s001.tif]

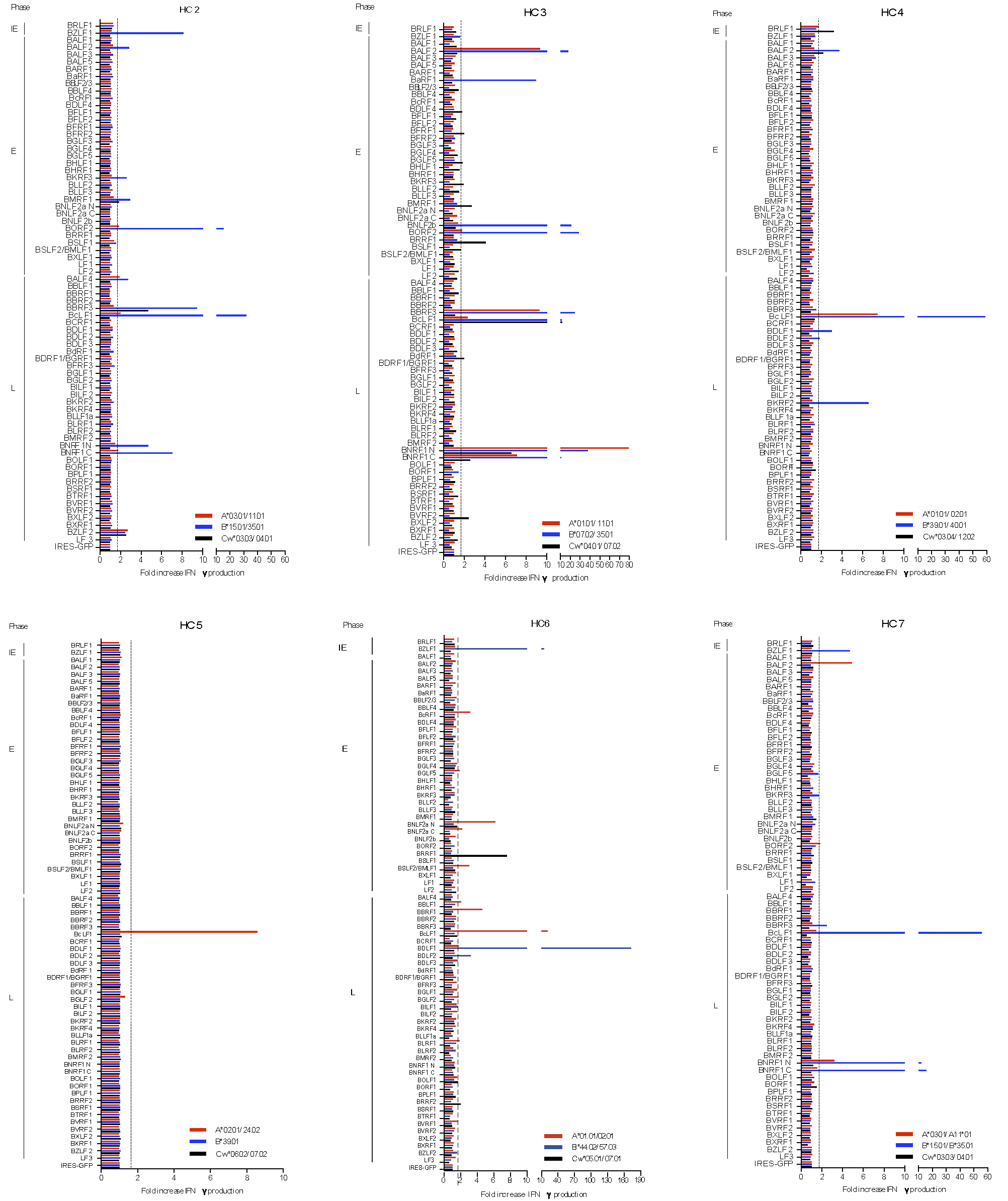

Supplement: S2 Fig — Data are presented as in Fig 2. (TIF) [file ppat.1007110.s002.tif]

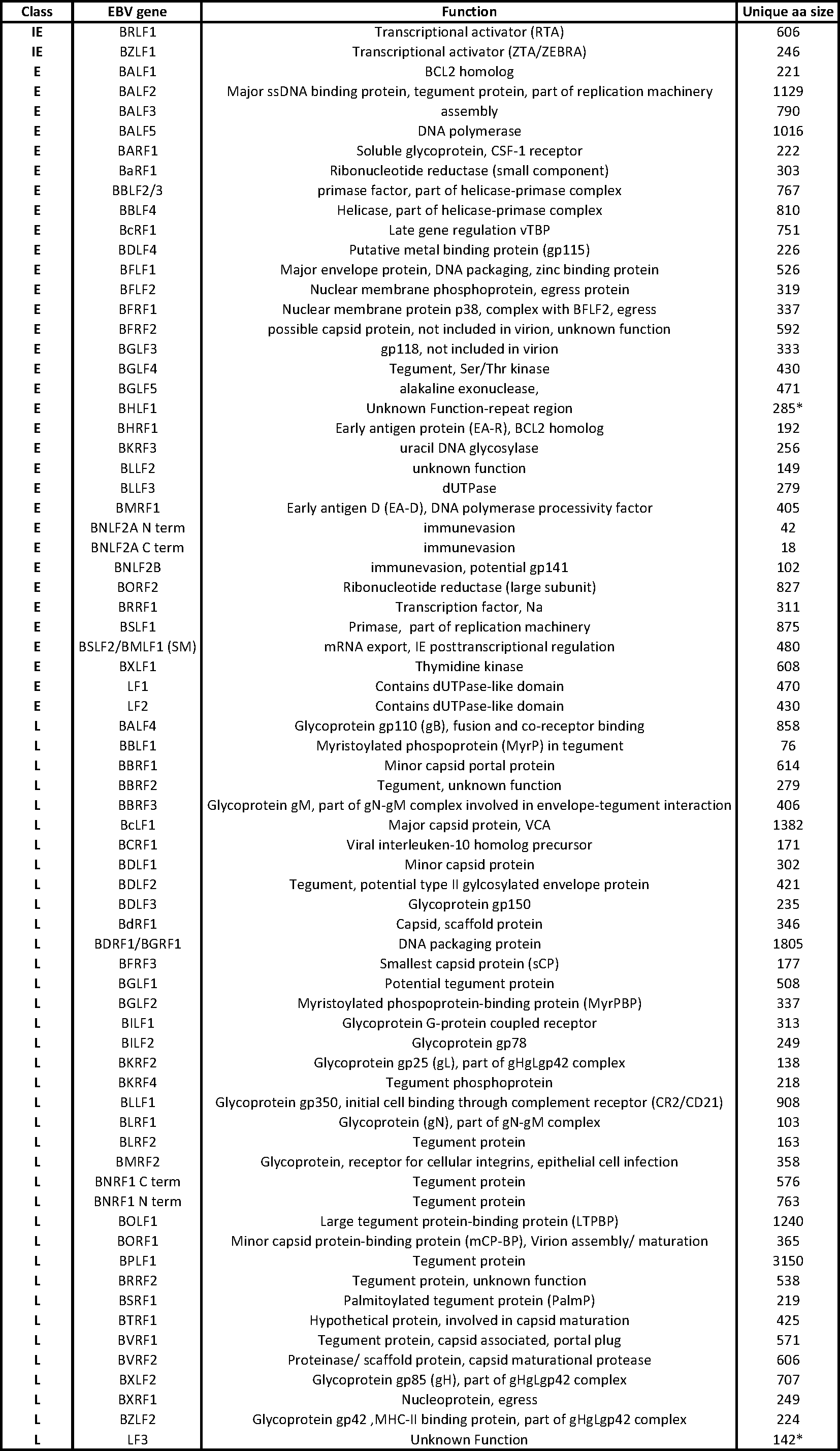

Supplement: S1 Table — The genes are arranged in IE, E and L categories and shown alongside the function (where known) and size (number of amino acid residues) of their protein products. * BHLF1 and LF3 contain many repeat domains and their sizes have been reduced to reflect their unique amino acid content. ** Two genes, BNLF2a and BNRF1, were expressed as separate N-terminal and C-terminal fragments. (TIF) [file ppat.1007110.s003.tif]

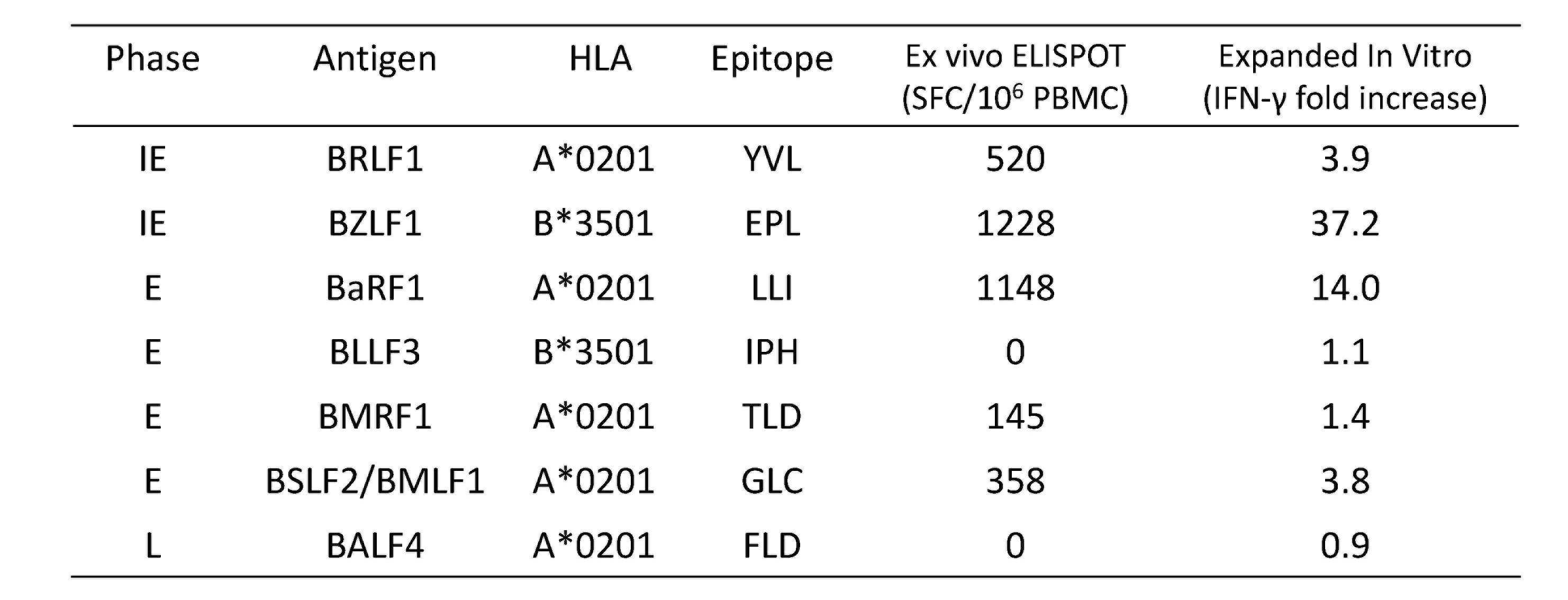

Supplement: S2 Table — PBMCs from HC1 were screened ex vivo for reactivity against defined HLA/peptides from 2 IE, 4 E and 1 L phase lytic antigens by IFN-γ ELISPOT. Results are shown as the mean number of spot-forming cells (SFC) per million PMBCs from replicate wells after subtraction of baseline reactivity to DMSO. In parallel, recognition of the relevant HLA/antigen combinations by in vitro expanded effector preparations from HC1 are shown as fold-increases in IFN-γ production over the GFP control vector background. Note that the smallest of the in vitro-expanded responses, against BMRF1, was reproducible but fell below the stringent 1.7-fold cut-off. (TIF) [file ppat.1007110.s004.tif]
