# Supplementary material for: Proteome-wide analysis of CD8+ T cell responses to EBV reveals differences between primary and persistent infection
Source: PLoS Pathog. 2018 Sep 24;14(9):e1007110. doi: 10.1371/journal.ppat.1007110 (PMC6171963; doi:10.1371/journal.ppat.1007110)
Supplement: S3 Table — Data are compiled for all the individual HLA-A, HLA-B and HLA-C alleles possessed by study donors. For each allele, the Table records the number of allele-positive donors, those antigens through which an allele-restricted response was observed, and the donors who made such a response. Shading identifies “prevalent” responses, i.e cases where more than half the donors with the relevant allele made a response to the specific allele/antigen combination. (PDF) [file ppat.1007110.s005.pdf]

| HLA A allele | protein | responding IM donors | responding HC donors |
|---|---|---|---|
| A*01.01 (7 donors) | BZLF1 | IM243, IM249 | |
| | BGLF4 | IM249 | |
| | BSLF2/BMLF1 (SM) | IM249 | |
| | BORF2 | IM217, IM243, IM249 | |
| | BBRF1 | IM217 | HC6 |
| | BPLF1 | IM249 | |
| A*02.01 (8 donors) | BRLF1 | IM84, IM223, IM239, IM249 | HC1, HC4 |
| | BALF1 | IM249 | |
| | BaRF1 | IM84, IM223, IM239, IM249 | HC1 |
| | BBLF2/3 | IM223 | |
| | BFRF2 | IM249 | |
| | BGLF4 | IM84 | |
| | BKRF3 | IM84, IM223, IM249 | |
| | BMRF1 | IM84, IM223, IM249 | |
| | BORF2 | IM84, IM223, IM249 | |
| | BRRF1 | IM84, IM223 | |
| | BSLF2/BMLF1 (SM) | IM84, IM249 | HC1, HC6 |
| | BXLF1 | IM249 | |
| | BALF4 | IM84, IM223, IM249 | |
| | BBRF3 | IM223 | |
| | BcLF1 | IM84, IM223, IM239, IM249 | HC1, HC4, HC5, HC6 |
| | BCRF1 | IM84 | |
| | BdRF1 | IM249 | |
| | BLRF1 | IM84 | |
| | BNRF1 C term | IM223, IM249 | |
| | BORF1 | IM84, IM223 | |
| A*03.01 (3 donors) | BRLF1 | IM269 | |
| | BZLF1 | IM217 | |
| | BALF2 | | HC7 |
| | BALF4 | | HC2 |
| | BVRF2 | IM217 | |
| | BZLF2 | | HC2 |
| A*11.01 (4 donors) | BZLF1 | | HC1 |
| | BALF2 | | HC3,HC7 |
| | BALF4 | | HC2 |
| | BVRF2 | IM217 | |
| | BORF2 | IM269 | HC1, HC2, HC3, HC7 |
| | BBRF3 | | HC3 |
| | BcLF1 | | HC2, HC3 |
| | BNRF1 N term | | HC1, HC3, HC7 |
| | BNRF1 C term | | HC2, HC3, |
| | BZLF2 | | HC2 |
| A*23.01 (1 donor) | BZLF1 | IM243 | |
| | BBLF4 | IM243 | |
| | BFRF1 | IM243 | |
| | BORF2 | IM243 | |
| | BBRF3 | IM243 | |
| A*24.02 (2 donors) | BZLF1 | IM223 | |
| | BALF1 | IM223 | |
| | BFRF1 | IM223 | |
| | BORF2 | IM223 | |
| | BcLF1 | IM223 | HC5 |

| HLA B allele | protein | responding IM donors | responding HC donors |
|---|---|---|---|
| B*07.02 | BALF1 | IM217 | |
| (2 donors) | BALF2 | IM217 | |
| | BaRF1 | IM217 | HC3 |
| | BBLF2/3 | IM217 | |
| | BFRF1 | IM217 | |
| | BGLF4 | IM217 | |
| | BLLF3 | IM217 | |
| | BNLF2b | | HC3 |
| | BORF2 | IM217 | HC3 |
| | BcLF1 | IM217 | HC3 |
| | BPLF1 | IM217 | |
| B*08.01 | BRLF1 | IM249 | |
| (5 donors) | BZLF1 | IM217, IM223, IM239, IM243, IM249 | |
| | BALF2 | IM217 | |
| | BBLF2/3 | IM249 | |
| | BMRF1 | IM223, IM243 | |
| | BORF2 | IM243 | |
| | BSLF2/BMLF1 (SM) | IM249 | |
| | BXLF1 | IM249 | |
| | BPLF1 | IM223 | |
| | BVRF2 | IM249 | |
| B*15.01 | BZLF1 | IM84 | HC2,HC7 |
| (3 donors) | BALF2 | IM84 | |
| | BGLF5 | IM269 | HC7 |
| | BSLF2/BMLF1 (SM) | IM84 | |
| | BcLF1 | IM84 | HC2, HC7 |
| | BDRF1/BGRF1 | IM84 | |
| | BNRF1 N term | | HC7 |
| | BNRF1 C term | | HC7 |
| | BRRF2 | IM84 | |
| | BZLF2 | | HC2 |
| B*35.01 | BZLF1 | IM269 | HC1, HC2, HC7 |
| (4 donors) | BALF2 | | HC2, HC3 |
| | BBLF2/3 | IM269 | |
| | BBLF4 | IM269 | |
| | BKRF3 | IM269 | HC2, HC7 |
| | BMRF1 | IM269 | HC1, HC2 |
| | BORF2 | IM269 | HC1, HC2 |
| | BALF4 | | HC2 |
| | BBRF3 | IM269 | HC1, HC2, HC3, HC7 |
| | BcLF1 | IM269 | HC1, HC2, HC3, HC7 |
| | BNRF1 N term | | HC1, HC2, HC3, HC7 |
| | BNRF1 C term | | HC2, HC3 |
| | BZLF2 | | HC2 |
| B39.01 | BALF2 | | HC4 |
| (2 donors) | BcLF1 | | HC4 |
| | BDLF1 | | HC4 |
| B*40.01 | BRLF1 | IM223 | |
| (2 donors) | BaRF1 | IM223 | |
| | BBLF2/3 | IM223 | |
| | BGLF5 | IM223 | |
| | BORF2 | IM223 | |
| | BSLF2/BMLF1 (SM) | IM223 | |
| | BRRF1 | IM223 | |
| | BXLF1 | IM223 | |
| | BcLF1 | IM223 | |
| | BKRF2 | IM223 | HC4 |
| | BNRF1 N term | IM223 | |
| | BNRF1 C term | IM223 | |
| | BSRF1 | IM223 | |
| | BVRF1 | IM223 | |
| B*44.02 | BZLF1 | IM84 | HC1, HC6 |
| (3 donors) | BHRF1 | IM84 | |
| | BBRF3 | | HC1 |
| | BDLF1 | | HC1, HC6 |
| | BDLF2 | | HC6 |
| B*44.03 | BZLF1 | IM243 | |
| (1 donor) | BMRF1 | IM243 | |
| | BORF2 | IM243 | |
| B*51.01 (1 donor) | - | | |
| B*55.01 | BORF2 | IM249 | |
| (1 donor) | BXLF1 | IM249 | |
| | BNRF1 C term | IM249 | |
| | BPLF1 | IM249 | |
| B*57.03 (1 donor) | - | | |

| HLA C allele | protein | responding IM donors | responding HC donors |
|---|---|---|---|
| C*03.03 | BRLF1 | IM249, IM269 | |
| (3 donors) | BZLF1 | IM249 | |
| | BALF2 | IM249, IM269 | |
| | BGLF4 | IM249 | |
| | BMRF1 | IM249, IM269 | HC2 |
| | BBRF1 | IM249 | |
| | BBRF3 | IM249 | HC2 |
| | BVRF2 | IM249 | |
| | BZLF2 | | HC2 |
| C*03.04 | BALF2 | IM84, IM223 | |
| (3 donors) | BBLF2/3 | IM223 | |
| | BBLF4 | IM84 | |
| | BMRF1 | IM84, IM223 | |
| | BBRF3 | IM223 | |
| C*04.01 | BALF2 | IM243 | |
| (5 donors) | BDLF4 | | HC3 |
| | BKRF3 | | HC3 |
| | BMRF1 | IM243, IM269 | HC1, HC3 |
| | BORF2 | IM243 | |
| | BNRF1 N term | | HC3 |
| | BNRF1 C term | | HC3 |
| | BZLF2 | | HC2 |
| C*05.01 | BBLF2/3 | IM84 | |
| (3 donors) | BBLF4 | IM84 | |
| C*06.02 | - | | |
| (1 donor) | | | |
| C*07.01 | BORF2 | IM243 | |
| (6 donors) | BRRF2 | IM223 | |
| C*07.02 | BFRF1 | | HC3 |
| (3 donors) | BcLF1 | | HC3 |
| | BdRF1 | | HC3 |
| C*12.02 | BRLF1 | | HC4 |
| (1 donor) | BALF2 | | HC4 |
